# Can I have your data? Recommendations and practical tips for sharing neuroimaging data upon a direct personal request

**DOI:** 10.1162/imag_a_00508

**Published:** 2025-03-19

**Authors:** Anita S. Jwa, Martin Norgaard, Russell A. Poldrack

**Affiliations:** Department of Psychology, Stanford University, Stanford, CA, United States; Department of Computer Science, University of Copenhagen, Copenhagen, Denmark; Molecular Imaging Branch, National Institute of Mental Health (NIMH), Bethesda, MD, United States

**Keywords:** neuroimaging, data sharing, data use agreement, regulatory analysis

## Abstract

Sharing neuroimaging data upon a direct personal request can be challenging both for researchers who request the data and for those who agree to share their data. Unlike sharing through repositories under standardized protocols and data use/sharing agreements, each party often needs to negotiate the terms of sharing and use of data case by case. This negotiation unfolds against a complex backdrop of ethical and regulatory requirements along with technical hurdles related to data transfer and management. These challenges can significantly delay the data-sharing process, and if not properly addressed, lead to potential tensions and disputes between sharing parties. This study aims to help researchers navigate these challenges by examining what to consider during the process of data sharing and by offering recommendations and practical tips. We first divided the process of sharing data upon a direct personal request into six stages: requesting data, reviewing the applicability of and requirements under relevant laws and regulations, negotiating terms for sharing and use of data, preparing and transferring data, managing and analyzing data, and sharing the outcome of secondary analysis of data. For each stage, we identified factors to consider through a review of ethical principles for human subject research; individual institutions’ and funding agencies’ policies; and applicable regulations in the U.S. and E.U. We then provide practical insights from a large-scale ongoing neuroimaging data-sharing project led by one of the authors as a case study. In this case study, PET/MRI data from a total of 782 subjects were collected through direct personal requests across seven sites in the USA, Canada, the UK, Denmark, Germany, and Austria. The case study also revealed that researchers should typically expect to spend an average of 8 months on data sharing efforts, with the timeline extending up to 24 months in some cases due to additional data requests or necessary corrections. The current state of data sharing via direct requests is far from ideal and presents significant challenges, particularly for early career scientists, who often have a limited time frame—typically 2 to 3 years—to work on a project. The best practices and practical tips offered in this study will help researchers streamline the process of sharing neuroimaging data while minimizing friction and frustrations.

## 1 Introduction

There has been growing interest in data sharing in the field of neuroimaging, and it has become an essential part of research practice (Breeze et al., 2012;Choudhury et al., 2014;Giehl et al., 2024;Mennes et al., 2013;Poldrack & Gorgolewski, 2014;Poline et al., 2012). Sharing neuroimaging research data enables validation of research findings, strengthens the analyses by combining multiple datasets for properly powered sample sizes, and increases the potential for data to contribute to new scientific discoveries. It also enhances public trust in neuroimaging research by fostering transparency and accountability. The increase in the size of shared neuroimaging data has fueled a surge in secondary analyses of the data. This, in turn, has accelerated advancements in the field, leading to a wealth of peer-reviewed publications (Milham et al., 2018).

Initially, data sharing occurred primarily between individual researchers by requesting it directly from the researchers who collected the data. But for the last few decades, numerous data repositories have been developed around the world, becoming the main avenue to share neuroimaging research data. Funding agencies (National Institutes of Health, 2020a) and academic journals (e.g.,Nature, n.d.;Imaging Neuroscience, n.d.) also recommend researchers deposit the data collected as part of a funded/published study in established repositories to streamline data sharing through standardized protocols. Sharing through data repositories would also improve FAIRness (Findability, Accessibility, Interoperability, and Reusability) of shared data (Wilkinson et al., 2016).

Many of the existing repositories are developed as designated archives for large-scale national/multinational research initiatives backed by private/government funding. Some of them cater to datasets collected from specific populations, such as patients with certain clinical conditions (e.g., Alzheimer’s Disease Neuroimaging Initiative (ADNI)) or individuals in certain age groups (e.g., Adolescent Brain Cognitive Development (ABCD) or Human Connectome Project (HCP) Young Adult). Others focus on particular data types, such as resting-state MRI (International Neuroimaging Data-Sharing Initiative (INDI)). The level of access control and restrictions on secondary data use varies greatly across these repositories (Jwa & Poldrack, 2022). This spectrum ranges from fully open access (e.g., OpenNeuro), whereby data are publicly available for unrestricted downstream analysis (other than prohibition from reidentification), to restricted access, which requires users to have verified credentials and imposes limitations on secondary analyses other than pre-approved research projects.

Despite the growth of data repositories, a significant portion of data sharing in the field still takes place through direct communication between researchers (Giehl et al., 2024;Paret et al., 2022). This can be attributed to several factors. Informed consent obtained for the initial data collection may limit how the data can be shared and used in the future, restricting deposit of such data in repositories. Some researchers may also have privacy concerns, especially when handling highly sensitive or easily identifiable data at the individual subject level. Furthermore, preparing and maintaining data for sharing through repositories can be burdensome for small research programs due to the limited resources, such as funding and administrative support.

However, sharing neuroimaging data upon a direct personal request can be challenging both for researchers who request the data and for those who agree to share their data. Unlike sharing through repositories under standardized protocols, each party often needs to negotiate the terms of sharing and use of data case by case. This negotiation unfolds against a complex backdrop of ethical and regulatory requirements along with technical hurdles related to data transfer and management. These challenges can significantly delay the data sharing process, and if not properly addressed, lead to potential tensions and disputes between sharing parties.

This article aims to help researchers navigate these challenges by exploring core considerations throughout the data-sharing process. It will also draw on practical insights from an ongoing case study led by one of the authors (MN), which involved extensive sharing of neuroimaging datasets from multiple research groups and institutions through direct personal requests.

## 2 Points to Consider when Sharing Human Neuroimaging Data upon a Direct Personal Request

Here, we divided the process of sharing data upon a direct personal request into six stages: (1) requesting data, (2) reviewing the applicability of and requirements under relevant laws and regulations, (3) negotiating terms for sharing and use of data, (4) preparing and transferring data, (5) managing and analyzing data, and (6) sharing the outcome of secondary analysis of data. For each stage, key factors to consider were identified from the ethical principles of human subject research, applicable laws and regulations, funding agencies’ data sharing policies, and institutional guidance on data sharing.

When sharing human neuroimaging data, researchers should adhere to international and regional ethical principles for human subject research—for example, the Declaration of Helsinki (World Medical Association, 2013) and the United States (US) Belmont Report (National Commission for the Protection of Human Subjects of Biomedical and Behavioral Research, 1979). These ethical principles commonly require subjects’ informed consent and voluntary participation, and researchers should respect subjects’ choices and uphold any conditions on future sharing and use of data laid out in the original consent. In addition, researchers have an ethical duty to conduct a systematic assessment of risks and benefits in a study and to minimize unnecessary harm to subjects. The major risk in data sharing is infringement of subject privacy and confidentiality of data. Sharing data could result in unauthorized disclosure of sensitive personal or health information, which could cause stigmatization and discriminatory harm to subjects. Researchers should implement rigorous privacy and security measures to share data and must remain vigilant regarding data misuse to prevent the potential harms.

A diverse array of domestic and regional laws and regulations govern the sharing and use of scientific data, posing important considerations for neuroimaging researchers. In this article, we will focus on the representative regulations in the US—the Federal Policy for the Protection of Human Subject (also known as the Common Rule) (U.S. Department of Health and Human Services (HHS), 2008) and the Health Insurance Portability and Accountability Act (HIPAA) (U.S. Department of Health and Human Services (HHS), 2003)—and in the EU—the General Data Protection Regulation (GDPR) (European Parliament & Council of the European Union (EU), 2016). Provisions specifically relevant to our analysis would be on the requirements for secondary use of research data and the standards for data processing to preserve privacy and confidentiality, which is referred to as data deidentification in the US and anonymization/pseudonymization in the EU.

Funding agencies’ data sharing policy provides another layer of guidance for researchers on sharing human neuroimaging data. Through its supplemental information, the US NIH’s new Data Management and Sharing (DMS) policy outlined the best practices and recommendations for how to protect subject privacy when sharing human research participant data, from deidentifying data to developing a data use agreement (National Institutes of Health, 2022). This supplemental information is not meant to be a guidance for regulatory compliance or to set forth binding obligations for investigators funded by the NIH, but it could be a useful reference for neuroimaging researchers.

Individual institutions also have their own policies for data sharing. For example, Stanford University, the institution with which the authors are currently (A.S.J. & R.A.P.) or formerly (M.N.) affiliated, provides internal guidelines particularly relevant to sharing data upon a personal request, such as when a data use agreement will be required and what elements should be included in the agreement (Office of Research Administration, Stanford University, 2023,^2024^).

### 2.1 STAGE 1: Requesting data

When a researcher identifies a dataset relevant to their future study, the first step to take is initiating a contact with the party who owns or is responsible for managing the data. This party could be an individual researcher(s), a research institution, or other private/public organization. If there is an associated publication from the identified dataset, it would be helpful to check the data availability statement for information on whom to contact. In this initial conversation, the requesting party should

- Briefly describe the goals and outline of the proposed secondary research.
- Inquire about the availability of the target dataset, more specifically the level and type of data available (e.g., whether the data are available at the individual level (raw data) or only in a derived form).
- Inquire about the availability of any associated metadata and other supporting documentation (e.g., codes or provenance records) that could facilitate interpretation and analysis of the data.

Our analysis of the later stages of data sharing primarily focuses on the United States and the European Union. However, different countries and regions can have significantly varying perspectives on data sharing. For instance, collecting research data in certain locations only to export them to higher-income countries in the Global North can be perceived as a form of research colonialism. Data requesters should remain mindful of these differing viewpoints and respect local concerns regarding data sharing.

Upon receipt of the request for data sharing, the owner or manager of the dataset should first verify the requester’s credentials to ensure the legitimacy of the request. In line with the ethical principles for human subject research, particularly the principle of respect for autonomy, it is critical to check whether any limitations on sharing and secondary use of data exist in the original informed consent. Likewise, following the principle of beneficence and justice, the owner or manager of the dataset would also want to consider if the proposed secondary analysis on shared data may cause harm to data subjects and what the rigor and social value of the proposed reuse are (Hendriks et al., 2022). In addition, it may be beneficial to verify whether an existing agreement for data sharing between the institutions of the involved parties is already in place and whether it would adequately cover the sharing of the requested data. If such an agreement exists, it could potentially save significant time for the parties involved.

### 2.2 STAGE 2: Reviewing the applicability of and requirements under relevant laws and regulations

Once both parties have agreed to share the data, the next stage is reviewing the applicability of and requirements under relevant laws and regulations. As privacy has increasingly become a global imperative, the regulatory landscape around data sharing has been rapidly evolving (Eke et al., 2021;Reer et al., 2023). In this section, we will provide a brief overview of regulatory regimes in the US and EU, particularly regarding secondary research on shared data. The principal factor determining the applicability of relevant laws and regulations is the identifiability of the shared data.

#### 2.2.1 United States

Most human subject research conducted within US academic institutions is governed by the Common Rule, which outlines federal protections for human subjects in research (U.S. Department of Health and Human Services (HHS), 2008).^*^Under the Common Rule, secondary research with identifiable private information is considered human subject research and subject to the two main requirements of the Rule, informed consent and IRB review, unless it falls under one of the exemptions.^†^Here, identifiable private information means “information for which the identity of the subject is or may readily be ascertained by the investigator (45 CFR §46.102 (e)(5)).” The Rule does not define the term “readily ascertainable,” but the definition of identifiability might change in the future as the 2018 revision to the Rule requires federal departments and agencies to reexamine the meaning of identifiable private information every 4 years to account for technological developments (45 CFR §46.102 (e)(7)).

On the other hand, if the shared data are not individually identifiable, secondary research on the data is considered to not involve human subjects. As a result, researchers are not required to obtain new informed consent from the subjects or to undergo IRB review.^‡^The Rule does not provide specific methods for rendering data nonidentifiable, but the Office of Human Research Protections(OHRP) guidance (2008)states that data are not individually identifiable when they cannot be linked to specific individuals by the investigator(s) either directly or indirectly through coding systems. For example, data are considered nonidentifiable if data are coded*and*the key to decipher the code is destroyed or access to the key is strictly prohibited (e.g., through an agreement between the investigators and holder of the key, IRB-approved written policies and operating procedures for a repository or data management center, or other legal requirements).

One remaining question here is whether it is possible to share or conduct secondary research on nonidentifiable data outside the scope of the original consent. For instance, this could arise if the original consent promised that subjects’ data will not be shared for future research or will only be used for specific research purposes.^§^Although secondary research on nonidentifiable data is no longer subject to the Common Rule, meaning there is no regulatory violation even when the research is not compatible with the original consent, the original investigator still has an ethical obligation to honor the agreement made with the subjects (Meyer, 2018;Secretary’s Advisory Committee on Human Research Protections, 2011). Institutions can implement policies that empower their IRBs to review data sharing plans, and sharing or using data that were collected under a consent form that explicitly stated the data would not be shared or would only be used for limited purposes likely constitutes a protocol violation (Meyer, 2018). Researchers should always consult their IRBs before sharing data if subjects were promised otherwise (Meyer, 2018).

Another relevant major federal regulation in the US is the Health Insurance Portability and Accountability Act of 1996 (HIPAA) (U.S. Department of Health and Human Services (HHS), 2003). The HIPAA Privacy Rule aims to protect individually identifiable health information by limiting its uses and disclosures under certain circumstances (45 CFR §164.502). The protections under the Privacy Rule only apply to information held by a covered entity, such as a health plan and a health care provider (45 CFR §160.103); many universities are not considered covered entities and thus not subject to HIPAA, but this should be ascertained for each institution. Yet, at the same time, the Privacy Rule permits a covered entity to create information not individually identifiable following the two deidentification standards provided in the Rule. Once information has been deidentified, the Rule does not restrict sharing and secondary use of this information, as it is no longer considered protected information under the Rule.

The first method is formal determination by a qualified expert that the risk is very small that the information could be used to identify the individual subjects (45 CFR §164.514(b)(1)). The second one is the safe harbor method which requires removal of all the 18 direct HIPAA identifiers, such as names, specific geographical information (e.g., address); dates related to a subject (e.g., date of birth); and full-face photographic images and any comparable images, in the information. This method also requires no actual knowledge on the side of a covered entity that the information could be used alone or in combination with other information to identify an individual subject to whom the information pertains (45 CFR §164.514(b)(2)). HIPAA also recognizes a partially deidentified dataset that retains some direct identifiers, which is called a limited dataset (45 C.F.R. § 164.514(e)(2)). A limited dataset may be used and disclosed for research, health care operations, and public health purposes, but unlike fully deidentified data, it is required that a covered entity and the recipient of the dataset enters into a data use agreement promising specified safeguards for the protected health information within the dataset (45 C.F.R. § 164.514(e)(3), (4)).

Due to its simplicity, the Safe Harbor standard has been widely adopted by neuroimaging researchers to deidentify data, regardless of their HIPAA-covered entity status. Before sharing their neuroimaging data, researchers typically redact and code potentially identifying information in the data, such as subject names in image file headers or analysis pathnames. It is also a common practice to deface structural scans, as facial features reconstructed from these scans can be considered one of the HIPAA direct identifiers—a comparable image to facial photos—and used to reidentify subjects.

However, it is important to note that neuroimaging data deidentified under the HIPAA Safe Harbor standard may not necessarily be nonidentifiable under the Common Rule. As discussed above, data are nonidentifiable under the Common Rule if they are coded and any key to the code is either destroyed or inaccessible. If HIPAA-deidentified neuroimaging data retain a code to permit reidentification and there are no agreements, policies, or legal requirements that prohibit the release of the key, the data would likely be viewed as indirectly identifiable under the Common Rule. Therefore, a dataset that meets HIPAA deidentification criteria can still be considered identifiable under the Common Rule and subject to the Rule’s two requirements—informed consent and IRB review—unless one of the exemptions to the Rule applies.

#### 2.2.2 European Union

The General Data Protection Regulation (GDPR) in the EU is a comprehensive data privacy and security law in the EU that governs collection, processing, sharing, and storage of personal data (European Parliament & Council of the European Union, 2016). Personal data are defined as information relating to an identified or identifiable natural person (Art. 4(1)). A data controller is a party that determines the purposes and means of its processing, and a data processor is a third party that processes the data on the controller’s behalf. The GDPR has a broad scope, covering all entities that process personal data of subjects within the EU. This includes entities offering goods and services to or monitoring the behaviors of these individuals, even if the entities are based outside the EU.

However, GDPR’s protections do not apply to data for which the subject is not or can no longer be identifiable, referred to as anonymous data (Recital 26). The standards of identifiability of data under GDPR are more stringent than those under US regulations. Anonymization under the GDPR is an irreversible process whereby a natural person cannot be reidentified taking into account all the means likely reasonable to be used either by the data provider or recipient.^**^In contrast, pseudonymization involves processing personal data so that they cannot be attributed to a specific individual without additional information (e.g., redacting identifiers and replacing them with a code) (Art. 4 (5)). This is comparable to data deidentification in the US. Although sharing and secondary use of deidentified data are generally exempt from US regulations, such as the Common Rule and HIPAA, pseudonymized data still qualify as personal data under GDPR.

Therefore, neuroimaging data deidentified using HIPAA’s Safe Harbor method—removal of identifiers in the metadata through a coding system and defacing of structural scans—or classified as nonidentifiable under the Common Rule would, at best, be considered pseudonymized data under the GDPR’s definitions (Eke et al., 2021). In other words, secondary research on pseudonymized neuroimaging data is subject to the same requirements and limitations as those applied to the processing of personal data.

GDPR establishes basic principles relating to the processing of personal data (e.g., “lawfulness, fairness, and transparency” and “purpose limitation”) (Art 5. 1(a), (b)) and requires a lawful basis for processing, such as data subjects’ specific consent (Art. 6 (1)). Under GDPR, data subjects are entitled to a number of privacy rights, which are aimed at giving subjects greater control over their personal data, along with mechanisms to enforce these rights (Art. 77–84).

Sensitive data, such as health data, are classified as special categories of personal data and afforded heightened protections (Art. 9). In general, processing of sensitive data is prohibited; however, GDPR allows certain exemptions, including processing for research purposes (Art. 9(2)(j)), providing some flexibility within the regulation’s strict privacy safeguards. If needed, a data protection impact assessment (DPIA) must be conducted before processing sensitive data to identify, assess, and mitigate privacy risks, thereby minimizing potential negative impacts on individuals (Art. 35). This process should be guided by a data protection officer, a designated expert appointed by the data controller and processor to ensure compliance with the GDPR and other data protection regulations.

These exemptions require safeguards to protect the fundamental rights and the interests of the data subject, in accordance with Member State law (Art. 89 (1)). Such safeguards should ensure that appropriate technical and organizational measures are implemented (Art 89 (1)), and Member States may permit derogations, which refer to a partial relaxation of the application of a law or regulation in certain circumstances, thereby exempting certain data subject rights for the purposes of scientific research. (Art 89 (2)). Although there are substantial variations regarding what constitutes these safeguards across Member States, some common measures include pseudonymization, access control, appointment of a responsible person for the research, and security management (Kindt et al., 2021). In the context of sharing neuroimaging data, additional suggested measures include explicit informed consent to the sharing of personal data, independent ethical oversight by an ethics committee, mechanism for access control, and data use agreements (Reer et al., 2023).

If exemption requirements for processing sensitive data for research purposes are met—if appropriate safeguards are in place and the data protection officers of the involved parties determine that the sharing and processing comply with the GDPR and relevant EU member state derogations or interpretations—then the data can be shared under these terms. In 2022, the Data Governance Act was also enacted to further facilitate data sharing within the EU by enhancing the mechanisms to increase data availability and removing technical and organizational obstacles to data sharing. However, sharing data with countries outside the EU, particularly those not recognized as providing a level of data protection comparable to the GDPR, remains highly challenging (Peloquin et al., 2020;Reer et al., 2023).

### 2.2 STAGE 3: Negotiating terms for sharing and use of data

After reviewing the regulatory requirements, both parties will begin negotiating specific terms for sharing and secondary use of data. In the US, formal contractual control over sharing and secondary (and further downstream) use of data, such as a data use agreement, is not required for nonidentifiable data under the Common Rule or HIPAA-deidentified data. However, individual institutions may have policies that require a data use agreement for sharing these data in certain circumstances. Although primarily focusing on sharing data through a repository, the new NIH Data Management and Sharing Policy also recommends the use of data sharing/use agreement even when data are deidentified (National Institutes of Health, 2022). In the EU, a data use agreement is recommended, if not required, as one of the safeguards to protect data subjects for processing of personal data for secondary research (Reer et al., 2023;Staunton et al., 2022).

Data use agreements typically require institutional review due to the obligations and potential risks they impose on the university/hospital, including risks of civil liability, regulatory noncompliance, reputational harm, and compromised research principles (Mello et al., 2020). This review, conducted by university attorneys or contract administrators, can be intensive and lengthy. Contributing factors to delays include procedural inefficiencies, incomplete information, a lack of incentives or familiarity with academic practices on the part of data suppliers, and unresponsiveness from researchers (Mello et al., 2020).

This section outlines the factors to consider when developing terms for sharing and secondary use of data, whether as a part of a formal data use agreement or email/verbal agreement, which entails reviewing the essential elements of a data use agreement based on relevant regulations, policies, and templates.Table 1provides a summary of these factors.

**Table 1.** Factors to consider when negotiating terms of sharing and use of data

Define	• The institution(s) and individual(s) who will provide the data and who will receive and use the data ○ [GDPR] Roles and responsibilities of each parties following the terms under the regulation • The permitted and prohibited uses and disclosures of data ○ [GDPR] Legal basis of under which the data will be shared and processed
Specify	• Types of data and data elements to be shared • Metadata, other relevant data, and any associated documentation that will be made accessible to facilitate interpretation of the shared data • Standards to be applied to the data and metadata, such as data formats and data identifiers • The period during which the data will be accessed and used • Methods used to transfer the data and receive and access the data • Ownership and redistribution of derived data • Reporting obligations regarding research results or patentable materials • Publication expectations and authorship • Data disposition requirements
Include	• Requirements to implement appropriate safeguards to transfer, receive, and use the data • Prohibition against reidentification or recontacting subjects • Flow-down terms and restrictions on further sharing and use of data, along with responsibilities regarding privacy and confidentiality • Data breach reporting and incident mitigation requirements • Reimbursement of the costs associated with data sharing • Indemnification clause
Describe	• Data deidentification requirements and methods used to deidentify data ○ [GDPR] Where the data lie in the spectrum from anonymous to pseudonymous as defined under the regulation • Tools and software needed to access and manipulate data and how these tools can be accessed
Confirm	• Institutional oversight and review on the risks of data sharing, limitations on sharing in original informed consent, and appropriate privacy and security protections • Compliance with relevant laws, rules, and regulations, as well as all applicable professional standards

First, HIPAA’s provisions on limited datasets serve as a useful reference for what should be included in a data use agreement (45 C.F.R. § 164.514(e)(4)(ii)). Under HIPAA, a data use agreement between the covered entity and the recipient of the limited dataset must establish what are permitted uses and disclosures of the dataset and who is permitted to use or receive the dataset. The recipient must agree not to use or further disclose the dataset beyond what the agreement allows. In addition, the agreement should include requirements to implement appropriate safeguards to prevent unauthorized use or disclosure of the dataset and to report any unauthorized use or disclosure of the dataset. Some of these safeguards might include establishing a comprehensive risk-based security, privacy, and compliance framework; restricting the location of data to secure facilities accessible only by authorized personnel; implementing technical access controls; encrypting data; deploying firewalls; prohibiting unauthorized copying or downloading; conducting security monitoring to detect potential vulnerabilities and intrusions; and specifying protocols for record retention and destruction (Rosati, 2022). The agreement should also ensure that any agents to whom the recipient provides the limited dataset agree to the same restrictions and conditions. Finally, the agreement should include prohibition against recontacting or reidentifying data subjects.

For sharing data subject to the GDPR, there are several key factors to consider when negotiating the terms between the involved parties. Each party’s roles and responsibilities must be clearly defined in the data use agreement, in accordance with the terms under the GDPR. For example, parties may agree to serve as a “data controller” or a “data processor,” and they will assume the associated risks accordingly. The agreement should also identify the legal basis under which the data will be shared and processed. If informed consent is the legal basis, the data use agreement should include details on the data subject’s right to withdraw consent, as well as the subsequent steps to be taken if consent is withdrawn. In addition, the data use agreement should clarify whether the data are considered anonymous or pseudonymous under the regulation, specify the permissible secondary uses of the data, and address the risks associated with applying broad consent for such secondary uses (Peloquin et al., 2020). Limitations on the reuse of existing data should also be discussed, as these constraints can significantly influence whether, and to what extent, the data requester can carry out their intended research.

Individual institutions’ policies may impose additional terms for sharing data for secondary research. For example, Stanford University, a US research institution, provides a detailed decision tree to determine whether a data use agreement is needed for sharing data (Office of Research Administration, Stanford University, 2023), along with a list of core elements of such an agreement (Office of Research Administration, Stanford University, 2024). Similar to HIPAA’s requirements, the parties should first define the institution(s) and individual(s) who will provide and receive the data, as well as the permitted and impermissible uses and disclosures of the data. The agreement should also ensure that data security safeguards to transfer, receive, and use the data are in place; prohibit reidentification and recontacting subjects; and include data breach reporting and incident mitigation requirements. To facilitate data sharing, Stanford further recommends specifying the types of data and a list of the specific data elements to be shared; the period during which the data will be accessed and used; methods to transfer the data; flow-down restrictions and terms; data disposition requirements; and data ownership (and specific rights retained by the owner). Finally, the policy suggests the agreement address reporting obligations regarding research results or patentable materials and publication expectations.

Furthermore, funding agencies might have their own policies and guidelines for data sharing, including requirements for a data use agreement. For instance, the NIH Data Management and Sharing Policy, which aims to reinforce the NIH’s longstanding commitment to data sharing, requires submission of and compliance with data management and sharing plans for research funded or conducted by NIH that results in the generation of scientific data (National Institutes of Health, 2020a). In a supplemental information to the Policy, the NIH outlined best practices for addressing privacy considerations to meet the broader ethical duty to protect research subjects beyond regulatory requirements (National Institutes of Health, 2022). NIH recommends the use of data sharing/use agreement when sharing data through a repository and suggests some key elements to protect subject privacy in such agreements. Agreements for submitting data to repositories should include assurances that an institutional oversight body has reviewed and considered the risks of data sharing, that sharing is consistent with informed consent, and that appropriate protections are in place. Agreements for data users should outline the responsibilities of all parties with access to the data and clearly inform the parties about data use limitations and their responsibilities regarding privacy and confidentiality. Both data-sharing and use agreements should explicitly address sharing limitations, prohibit attempts to reidentify or recontact participants, describe methods used to deidentify data, and include any relevant risk assessments.

In addition, certain components of data management and sharing plan could also be useful to consider (National Institutes of Health, 2020b). For example, the plan requires investigators to specify not just the types of data to be shared but also metadata, other relevant data, and any associated documentation that will be made accessible to facilitate interpretation of the shared data. Investigators should also describe tools and software needed to access and manipulate data and how these tools can be accessed. Standards to be applied to the data and metadata, such as data formats and data identifiers, should also be included in the plan. Although the suggested key elements for data use agreements and the requirements for data management and sharing plan under the NIH’s new policy are not specifically intended for sharing data upon a personal request, both parties would want to consider them when negotiating terms to better protect subject privacy and minimize uncertainties in sharing the data.

The Data Stewardship Committee of the Federal Demonstration Partnership (FDP) in the US—an association of federal agencies, research policy organizations, and academic research institutions to streamline the administration of federally sponsored research—has developed a Data Transfer and Use Agreement (DTUA) template that has been widely adopted among the research institutions in the US (Federal Demonstration Partnership, n.d.). This template consists of a basic agreement and additional data protection terms and conditions that can be added for different types of data and situations (deidentified human subject data, limited dataset, personally identifiable information governed by either Common Rule only or HIPAA). Some elements in the template that have not been highlighted in the regulation and policies discussed above include: reimbursement of the costs associated with preparation, compilation, and transfer of the data; ensuring the use of data in compliance with all applicable laws, rules, and regulations, as well as all applicable professional standards; review and/or delay of manuscripts or abstracts for publication to ensure that the data are appropriately protected; a disclaimer that the data is shared “as is,” with no warranties of any kind; and an indemnification clause where the requestor assumes all liability arising from the use, storage, disclosure, or disposal of the data.

Finally, Open Brain Consent (Bannier et al., 2021), an international initiative that aims to provide neuroimaging researchers with information about data sharing options and tools, has developed a template for data use agreements, specifically designed for researchers subject to the GDPR. The provisions in the templates mostly align with the elements reviewed above, including compliance with relevant laws and regulations, prohibition on reidentification of subjects, restrictions on redistribution of shared data, and requirement to acknowledge the data source in resulting publications. However, there is one provision that has not been discussed earlier but warrants attention regardless of the regulatory regime under which the data sharing will occur: the redistribution of secondary and derived data. Ownership and redistribution of derived data is a frequently contentious issue in neuroimaging data sharing (Open Brain Consent, n.d.). To prevent potential conflicts down the line, we strongly recommend that this issue be addressed during negotiations. Both parties should clarify what qualifies as derived data, who owns the derived data, and whether and how its redistribution will be restricted.

### 2.4 STAGE 4: Preparing and transferring data

Once both parties have agreed on the terms of sharing and secondary use of data, the actual process of sharing will begin. The data provider should prepare and transfer the data as agreed between the parties. Again, clarifying the specific terms of sharing during the negotiation phase will significantly reduce the time and effort required from both sides at this stage. All data, metadata, and other relevant information, such as code and scripts used to analyze the data, should be transferred or made accessible to the requester using the methods specified in the agreement. For example, when sharing individual-level raw MRI data, which can be as large as several gigabytes, the data may need to be transferred by allowing the requester to download them directly from a secure cloud server. If the data are to be deidentified in a particular way, the provider should ensure this is done before sharing. It is also common to use community-developed data formats, such as Brain Imaging Data Structure (BIDS) (Gorgolewski et al., 2016;Poldrack et al., 2024), especially for large-scale neuroimaging data sharing. Using standardized formats like BIDS can streamline the process of understanding the structure of the data and minimize misunderstandings, leading to fewer errors in downstream data analysis. If there is a specific data format that the parties agreed upon, the provider should organize data in compliance with that format before sharing. Additionally, it would also be essential to inform the requester of provenance of data, including any preprocessing steps applied to the data, to facilitate the data analysis. This latter point on informing the requester whether any preprocessing steps (such as defacing or skull stripping) were applied to the data is particularly important, as it will avoid miscommunication and limit downstream requests to fix or answer questions related to the data.

### 2.5 STAGE 5: Managing and analyzing data

After the data transfer is completed, the data are now in the requester’s hands, and it is time for the requester to conduct their own secondary analysis. While analyzing the data, the requester should manage them in accordance with the terms of the agreement. All required data security safeguards should be in place, and it is the requester’s duty to report any incidents of unauthorized—whether intentional or inadvertent—access, disclosure, or misuse of the data. The requester should also follow the agreed-upon mitigation process to minimize the impact and damage caused by such incidents. Any personnel involved in the data analysis on the requester’s side must comply with the responsibilities and restrictions outlined in the agreement, including the prohibition against reidentifying or recontacting subjects. It is not uncommon at this secondary analysis stage to identify subject-specific inconsistencies that may require further attention. This may include identification of subjects with extensive motion artefacts, structural abnormalities (such as enlarged ventricles) or small variations in scan protocol that were not identified or reported in the original study. Furthermore, if the data has been preprocessed prior to sharing such as skull stripping or bias field correction, modern state-of-the-art quality control tools may identify subjects where preprocessing for these subjects have failed. If the data requester identifies inconsistencies or errors in the data, we encourage the requester to be mindful about communicating this back to the data owner, and discuss an optimal strategy for moving forward (e.g., whether a given subject should be re-processed or removed from the sample).

### 2.6 STAGE 6: Sharing the outcome of the secondary analysis of data

Upon completion of the secondary data analysis, the outcomes, including any data derived from the analysis, should also be shared in accordance with the terms of the agreement. For example, it may be stated in the contract that “Results/Outcomes are owned by the Party that generates them” and that “each Party may use the Results/Outcomes of the Project for internal, non-commercial research and teaching purposes”, highlighting that secondary analysis outputs may be shared with others given that it is defined as non-commercial research. If the secondary analysis outputs are intended to be used for commercial purposes, the contract may include text such as “A Party may use the Results for commercial purposes or grant non-exclusive licenses if the other Party is given advance notice and fair and reasonable compensation. This will be governed by a separate agreement.”. The data requester might be obliged to report research findings and patentable materials to the provider. It is commonly required to acknowledge the provider in resulting publications, and the provider might be listed as a co-author in the publication(s), if agreed upon. After the specified period to access data, the data should be properly disposed of in compliance with the agreement.

## 3 Practical Tips from a Case Study

In an ideal world, the process of sharing data would proceed smoothly if all the factors discussed in the previous section are carefully considered at each stage of sharing. Both the requester and provider would be highly motivated to collaborate, and with strong support from the institutional offices on both sides, the required ethics review and regulatory oversight would be completed in a timely manner. The data would be prepared and transferred without any errors in formatting and preprocessing, accompanied by all the necessary information to aid data interpretations and analysis. However, in reality, data sharing is often, if not always, accompanied by various challenges. These can include hesitation from either party for sharing and negotiating the certain terms of sharing, unexpected delays in the institutional review process, technical or human errors in handling the data, and disputes over authorship and dissemination of the outcomes from the secondary research—issues that can sometimes escalate and become emotionally charged.

**Fig. 1.**
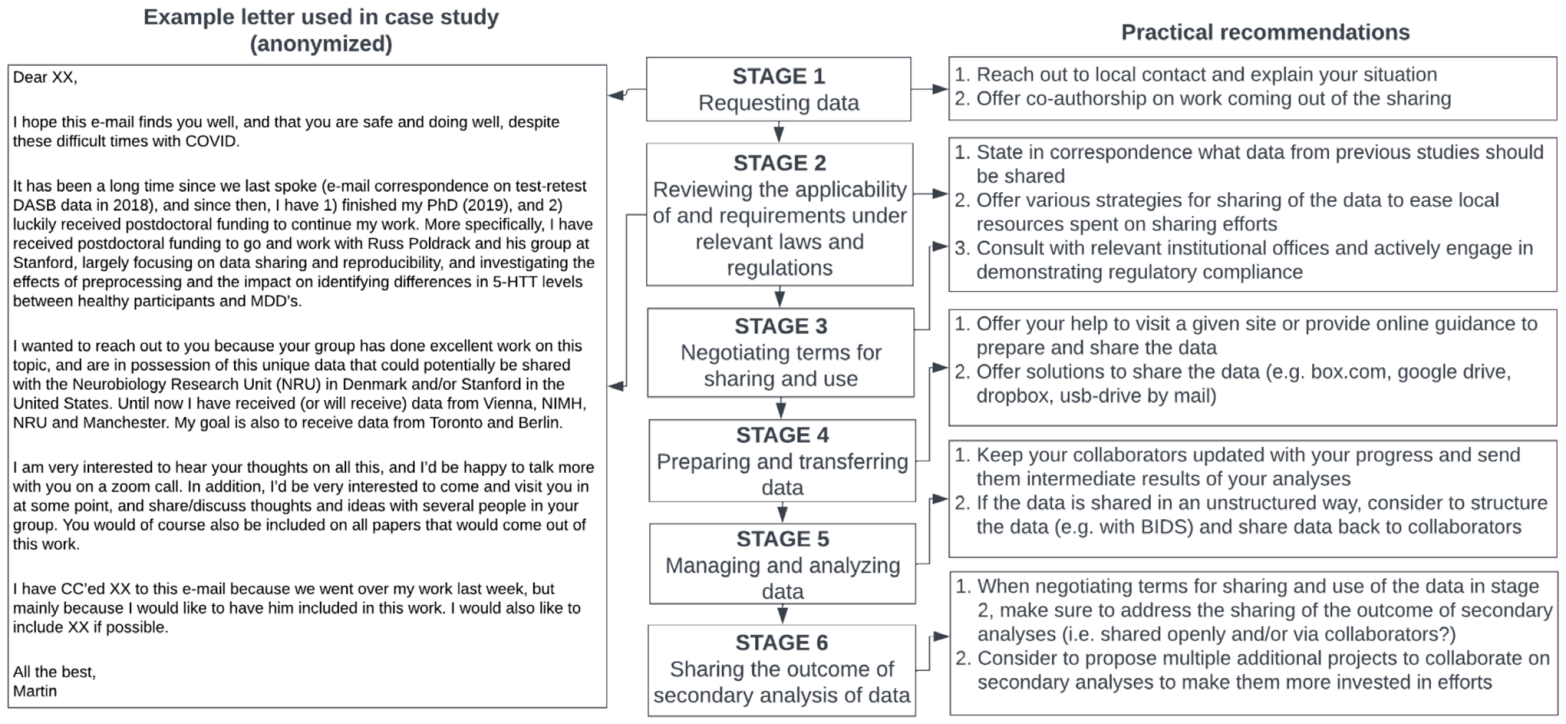
(Left) example letter used in case study to request data and facilitate sharing. This email was followed up by several pieces of correspondence and requests as indicated inTable 1. (Middle) Defining the six stages of sharing through direct personal requests (Right) Recommendations based on case study to have in mind throughout the six stages of sharing through direct personal request.

In this section, we will examine a large-scale neuroimaging data sharing project led by one of the authors (MN), as a case study, offering practical tips to facilitate sharing and secondary analysis of data while minimizing friction at each stage of data sharing (Fig. 1). This project aimed to collect a total of 782 subjects’ raw PET/MRI data and metadata from seven sites located in the USA, Canada, UK, Denmark, Germany, and Austria, through direct personal requests. The data consisted of several smaller independent studies investigating whether serotonin transporter (5-HTT) levels, as measured with PET, are abnormal in patients with Major Depressive Disorder (MDD) compared to healthy controls (HC) (e.g.,Cannon et al., 2007;Miller et al., 2013). All the requested data had previously been reported in peer-reviewed publications, with some of the data ranging back to 2006. Individual studies examining differences in 5-HTT levels between individuals with MDD and healthy controls (HC) have yielded inconsistent results, including both positive and null findings. These inconsistencies may be related to small sample sizes and variations in the analytical strategies employed. Therefore, the goal of the large-scale data sharing project led by MN was to address, with sufficient statistical power and with the same analysis strategy, whether there was a difference in 5-HTT levels between MDD’s and HC’s keeping the original research purpose intact.

In the STAGE 1 (Requesting data), one key takeaway from the case study is the importance of contacting the research team that owns data well in advance. As shown inTable 2, it took an average of 7.8 months (ranging from 2 to 12 months) per site to complete the data sharing. The amount of email correspondence varied from 1 to more than 100. Moreover, for some sites, additional requests were necessary to resolve inconsistencies or errors found during data processing (an average of seven requests across four sites), extending the sharing process up to 24 months. If possible, leveraging a personal contact for the data request could help reduce barriers to initiating a conversation with the data provider.

**Table 2.** Case study data on sharing through direct personal request, capturing the 7 sites spanning the countries of the US, Canada, UK, Denmark, Germany, and Austria.

Site	Status	Time from initial contact to receival/rejection	Amount of email correspondence	Number of requests to fix shared data
Site A	Reject	3 months	5	-
Site B	Received	6 months	39	6
Site C	Received	12 months	+100	20
Site D	Ongoing	12 months	14	-
Site E	Received	2 months	3	0
Site F	Received	12 months	32	2
Site G	Reject	Never responded	1	-

As of November 2024, four of the seven sites have currently shared their data, one site is ongoing, and two sites rejected sharing. Time from initial contact to receipt/rejection varied from 3 to 12 months. Amount of email correspondence varied from 1 to 100 +. Number of requests to fix inconsistencies/errors in shared data found during processing varied from 0 and up to 20.

For this case study, two out of the seven sites declined to share their data. Examples of reasons for rejecting to share data included “While I greatly appreciate the invitation, I am swamped with other commitments. Our system for grant funding in XX has become very chaotic and I have to concentrate my efforts on maintaining funding for upcoming projects”, “Those were the days of no databases (anywhere) and storing data on the hard drive of a single computer. While the data were held in XX’s lab, I’m not sure he still has the raw data (he can comment)”, and “The historical DASB data are not usable. There are issues around those data that made them untouchable.” In some circumstances, data sharing may be substantially restricted due to regulatory prohibitions. However, with thoughtful preparation and effort, most of the common reasons for data providers to reject a request can be addressed. While sharing data only with a single requester might be less burdensome than preparing it for repositories, the process can still be time-consuming and require additional resources. Moreover, a survey of neuroimaging researchers regarding open science practices found that a significant portion of the respondents (40%) had never learned how to share their data online (Paret et al., 2022). As suggested below, proposing to help and guide the data providers in preparing and transferring the data would significantly lower their burden. Other concerns, such as being scooped by other researchers or not receiving proper recognition for sharing data, may also contribute to a decision to withhold data, particularly for early career scientists. Offering co-authorship on publications resulting from the secondary analysis at the outset could help mitigate these concerns and encourage data providers to actively engage in data sharing.

Furthermore, to avoid missing information or inconsistencies in the data, it is highly recommended to be as specific as possible when requesting the data. The motivation from the data provider to share data may drop if this is not clear from the beginning, as local resources may be limited to curate and share the data. To accommodate this, the data requester may offer assistance in curating the data, or be flexible in terms of format the data comes in. For example, ideally the data should be curated following some standardized structure like BIDS; however, several sites do not have this implemented, meaning that metadata often exists in different formats and with different naming conventions (e.g., excel, csv etc). Furthermore, many labs also have their own preferred neuroimaging format for storing data (Analyze.img/.hdr or DICOM .dcm), and sometimes also with minimal preprocessing steps applied to the data such as skull stripping. Based on this, it is recommended, from the data requesting side, to be mindful and flexible about the current status of the data, to minimize the extra efforts required from the data provider to curate/convert data before sharing, especially if it does not impact the quality of the data or downstream analyses (e.g., converting analyze to nifti). If this is not taken into account, it may significantly affect the motivation to share the data from the data provider’s side, and based on resources, may prolong the data sharing time substantially.

When reviewing the regulatory requirements (STAGE 2), it is essential to consult with institutional offices, such as the Institutional Review Board (IRB) or privacy office at both sites, to ensure all regulatory requirements are met, especially in the case of sharing data across borders. Personnel at these offices, such as lawyers and ethics review panels, are often managing heavy workloads, so some delays in the review process should be anticipated. For example, for the case study, the terms for sharing of data between Copenhagen University Hospital and another European site had to be negotiated. However, at the time of the negotiation (early 2021), Copenhagen University Hospital was heavily overloaded with GDPR legal requests, so they had decided to outsource some of their legal responsibilities to an external legal company. It took 5 months to negotiate the terms for the data sharing, however, without Copenhagen University Hospital prioritizing to outsource their legal requests it is likely that the time for negotiation would have been substantially prolonged. Particularly regarding the privacy risk, these legal offices tend to be risk averse to protect the institutions’ interests, lacking the scientific and technical expertise to assess the risk, such as the adequacy of deidentification methods used. Therefore, the parties should be actively involved to educate the personnel to enable informed decision-making on regulatory compliance. Although it did not occur in any of the data-sharing transactions in the case study, cross-border data sharing may require permission from the funding agency that supported the original study, in addition to approvals from the relevant IRBs and privacy offices.

Aligned with the emphasis in STAGE 1, it is crucial to clearly specify the type and level of data, metadata, and other relevant information—such as code, analysis scripts, and data provenance—when negotiating the terms of sharing between the parties (STAGE 3). For example, in one instance of the case study, it was not until after the negotiation phase and data sharing phase that it was identified that a majority of participants were missing structural MRI data along with the PET data. Having structural MRI data available is crucial to carry out optimal PET preprocessing, because the MRI data is used for segmentation and normalization purposes. In the case of missing MRI data, the data may be deemed unusable or may need to go through suboptimal preprocessing to obtain reasonable results, however, it is not clear how and if it is valid to combine with the remaining optimally processed PET data.

As recommended earlier, the requester might want to propose various strategies for sharing the data to reduce the local resources required from the provider’s side. In the case study, all strategies for sharing the data were different, but were carried out based on suggestions from the data owner side, suggesting that flexibility to accommodate the needs/resources of the data owner may increase the chances of successful sharing. Finally, the ownership and redistribution of derived data should be addressed early in the process, as these issues could be a potential source of conflict. For example, in one of the cases from the case study, this was not discussed until after the analysis of the data, resulting in complications as the derived data from all the sites were intended to be shared openly after the analysis. Therefore, when it comes to sharing derived data, parties should clarify whether the data will be shared openly or only through restricted access. The tools and software needed to access and manipulate the shared data should also be outlined.

In terms of preparing and transferring the data (STAGE 4), having a reliable local contact directly involved in dealing with the data is crucial. Because data preparation on the provider’s side can take time, the requester should remain patient but can also take a proactive approach. Offering assistance to help facilitate the data preparation and sharing process, such as paying a visit to the provider’s site or providing online guidance to prepare and share the data, can be helpful. In addition, suggesting different solutions to transfer the data (e.g., box.com, Google drive, Dropbox, or USB-drive by mail) can streamline the transfer and ensure that potential delays are minimized. All these solutions to transfer data were suggested by the data owners to the data requester during the case study. If the data provider has already successfully shared data before, it is recommended to accept their solution to transfer the data, to minimize extra work efforts for the data provider.

While the data are being analyzed (STAGE 5), it is recommended to regularly update the provider with the progress in the secondary research and share any intermediate results to maintain a strong motivation from the data provider’s side. This is particularly important if additional requests are needed later, such as extra metadata or clarifying inconsistencies in the data. If the data were initially shared in an unstructured format, the requester should consider organizing the data according to a community standard, such as BIDS, and share the newly formatted data with the provider.

In the final stage of data sharing (STAGE 6: sharing the outcome of secondary analysis), the requester should share the derived data, if permitted under the agreement, and ensure that subsequent users comply with the flow-down terms of sharing. Depending on the outcome of the study, the requester may want to consider proposing multiple additional projects to further expand the collaboration, encouraging the provider to become more invested in time and effort. This approach can help strengthen the partnership and facilitate future research opportunities.

## 4 Conclusion

Data sharing has become an integral part of neuroimaging research. Over the past few decades, the volume and scope of shared neuroimaging data have expanded significantly, driving a wealth of secondary analyses that have enhanced the reproducibility of neuroimaging research and accelerated new discoveries about the human brain and its functions. Although the use of established repositories, which have standardized protocols and data sharing/use agreements, is encouraged, some researchers prefer to share data through personal requests due to a variety of reasons. However, sharing data through direct communication between researchers can be a daunting process. Researchers need to negotiate every detail of the terms of sharing by themselves, addressing complex ethical and regulatory hurdles, and often wrestle with technical challenges of transferring and managing large neuroimaging datasets. This process requires careful coordination and a great deal of patience from both parties.

In this study, we suggested best practices for sharing data upon direct personal request by summarizing crucial factors to consider at each stage of the data sharing through a review of ethical, policy, and regulatory requirements. Growing concerns about data privacy have prompted rapid changes in data protection regulations. Governments and institutions worldwide are enacting stricter policies and regulations for data protection, which could substantially affect open science practices in neuroimaging. This evolving landscape demands heightened vigilance and compliance from researchers, making it essential to navigate both local and international regulations effectively for compliance, working closely with their institutional offices. We also offered practical tips based on a case study to help streamline the process of sharing data through direct requests while minimizing friction and frustration. The case study also revealed that researchers should typically expect to spend an average of 8 months on data sharing efforts, with the timeline extending up to 24 months in some cases due to additional data requests or necessary corrections. The current state of data sharing via direct requests is far from ideal and presents significant challenges, particularly for early career scientists, who often have a limited time frame—typically 2 to 3 years—to work on a project, making such delays especially risky in terms of their research progress and career prospects.

## Data Availability

No new data were created or analyzed to support this study.

## Footnotes

* The Rule applies to human subject research conducted or supported by a federal department or agency adopting the Rule. Institutions engage in research funded by a Common Rule department must comply with the Rule’s requirements, but many have voluntarily extended the scope of their commitment to cover all human subject research, regardless of funding source (Meyer, 2020).

† Secondary research on identifiable private information could be exempted from the Common Rule requirements, for example, if the data source is publicly available (45 CFR §46.104 (4)(i)) or the investigator recorded the information in such a manner that the identity of human subjects cannot be readily ascertained and the investigator does not contact or reidentify subjects (45 CFR §46.104 (4)(ii)). In addition, when broad consent is obtained for the storage, maintenance, and secondary research use of identifiable information, secondary research on the information does not require additional consent, as long as other conditions are met, including limited review by an IRB (45 CFR §46.104(8)).

‡ It is important to note that some IRBs might impose more strict standards in terms of the identifiability of data than those outlined in the Common Rule. Therefore, it’s essential to consult with the IRB to ensure compliance, even if the data appears non-identifiable under the Common Rule.

§ After the revision of the Common Rule in 2018, researchers are required to include a notice in the informed consent form stating whether subjects’ data might be stripped of identifiers and used for future research or distributed to another researcher for future studies without additional informed consent from the subjects (45 CFR §46.116 (b)(9)).

** All objective factors, such as the costs of and the amount of time required for identification, should be considered to ascertain whether means are reasonably likely to be used to identify the natural person, given the available technology at the time of the processing and technological developments.
